# Supplementary material for: Learning to detect AI texts and learning the limits
Source: PLoS One. 2025 Oct 15;20(10):e0333007. doi: 10.1371/journal.pone.0333007 (PMC12527182; doi:10.1371/journal.pone.0333007)
Supplement: S2 File — All data and scripts used in the study, some additional descriptive statistics, scalable formate figures. (ZIP) [file pone.0333007.s002.tgz › data and scripts/OSF/scripts/analysis/Dependencies/id_vs_More_readable_is_human.pdf]

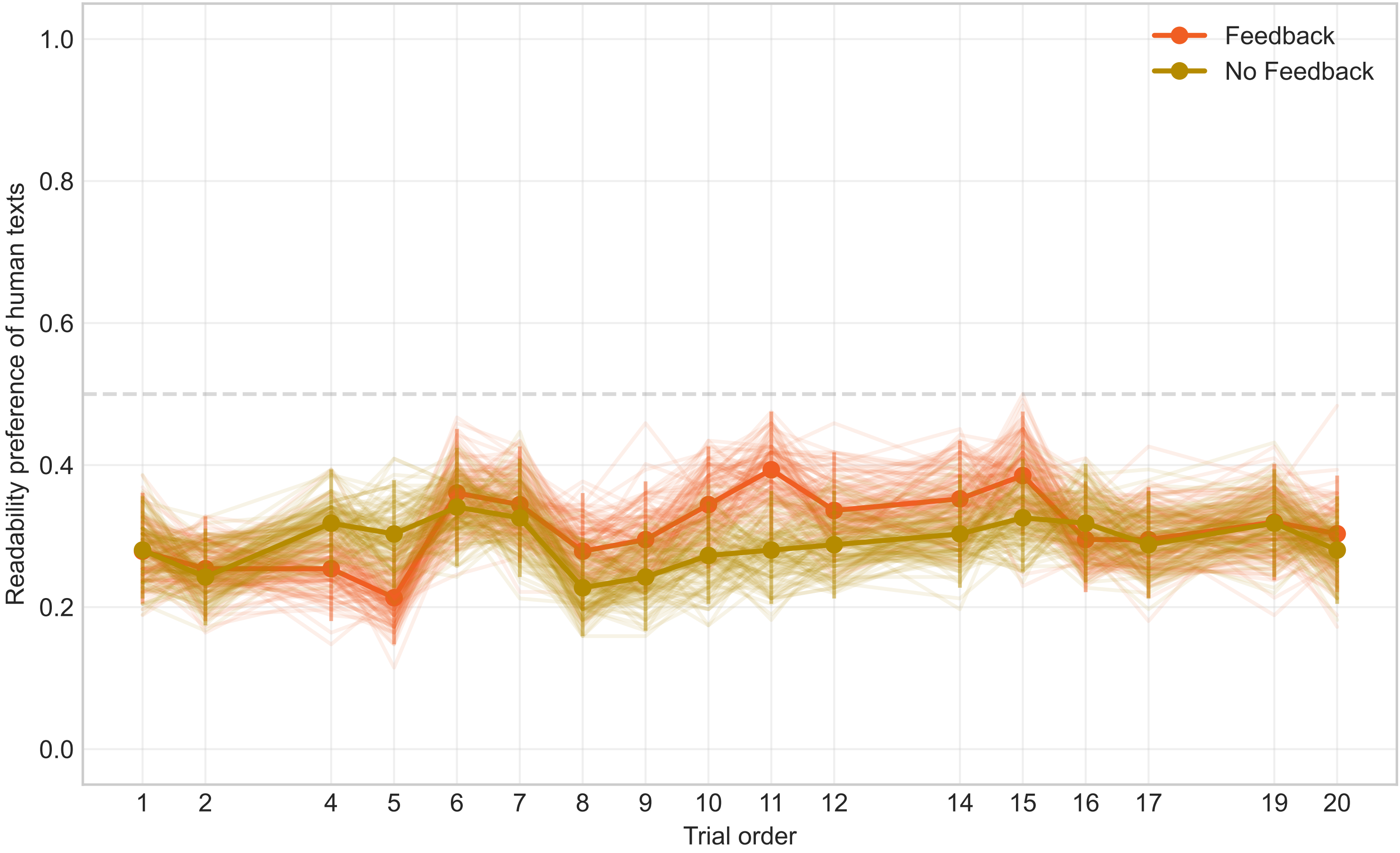
Feedback
No Feedback
Readability preference of human texts
Trial order
0.0
0.2
0.4
0.6
0.8
1.0
1
2
4
5
6
7
8
9
10
11
12
14
15
16
17
19
20
